# Radiation Therapy-Induced Cardiovascular Disease Treated by a Percutaneous Approach

**DOI:** 10.1155/2015/851624

**Published:** 2015-04-30

**Authors:** Luigi Fiocca, Micol Coccato, Vasile Sirbu, Angelina Vassileva, Giulio Guagliumi, Giuseppe Musumeci, Amedeo Terzi, Gianluca Canu, Elisa Cerchierini, Diego Cugola, Orazio Valsecchi

**Affiliations:** ^1^Cardiovascular Department, Papa Giovanni XXIII Hospital, 24127 Bergamo, Italy; ^2^Anesthesia and Intensive Care Department, Papa Giovanni XXIII Hospital, 24127 Bergamo, Italy

## Abstract

We report the case of a 51-year-old woman, treated with radiotherapy at the age of two years, for a pulmonary sarcoma. Subsequently she developed severe aortic stenosis and bilateral ostial coronary artery disease, symptomatic for dyspnea (NYHA III functional class). Due to the prohibitive surgical risk, she underwent successful stenting in the right coronary artery and left main ostia with drug eluting stents and, afterwards, transcatheter aortic valve replacement with transfemoral implantation of a 23 mm Edwards SAPIEN XT valve. The percutaneous treatment was successful without complications and the patient is in NYHA II functional class at 2 years' follow-up, fully carrying out normal daily activities.

## 1. Introduction

Cardiovascular diseases in patients who undergo thoracic radiotherapy represent a significant reason for long-term mortality. Vascular disease after radiotherapy usually occurs through an accelerated development of age related atherosclerosis, often involving coronary ostia, resulting in an increased risk of myocardial infarction or sudden cardiac death [1]. Early fibrosis, involving cardiac valves, is common after radiotherapy, with important prognostic consequences [2–4].

Surgery management of these patients is troublesome, due to anatomical sequelae of radiation exposure, with adherence and pulmonary fibrosis that increase the surgical risk.

Percutaneous treatment of coronary and structural disease is nowadays widely applied, representing a valid option for high surgical risk patients.

Transcatheter aortic valve implantation (TAVI) is increasingly popular as an alternative for symptomatic patients affected by severe aortic stenosis that are at high risk for surgery [5–7].

## 2. Case Report

A 51-year-old woman with pulmonary fibrosarcoma diagnosed at age 2, treated with repeated cycles of radiotherapy, presented with exertional dyspnea.

**Figure 1:**
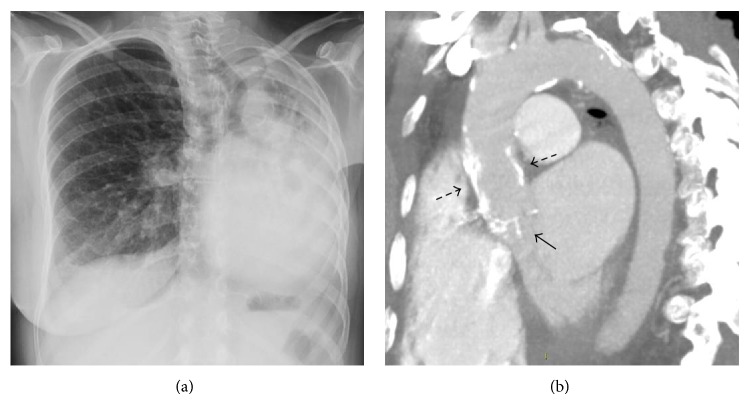
(a) Chest X-ray demonstrating left fibrothorax with ipsilateral mediastinum and trachea displacement. (b) CT scan demonstrating thickening and calcification of the aortic valvular leaflets (arrow) and diffuse calcification of the ascending aorta (dashed arrows).

She had late sequelae of radiotherapy including mediastinal and left pulmonary fibrosis, with severe restrictive lung disease (Figure 1). She underwent a cardiologic evaluation for worsening dyspnea, which revealed a markedly impaired functional capacity (NYHA III functional class). Transthoracic and transesophageal echocardiogram revealed mildly impaired left ventricular function (ejection fraction 45%), severe aortic stenosis (mean gradient 40 mmHg, indexed valve area 0.5 cm^2^/m^2^), and moderate aortic and mitral regurgitation. An angio-CT scan showed thickening and calcification of the aortic valve and a porcelain aorta, supporting the diagnosis of radiation-induced cardiovascular disease (Figure 1).

**Figure 2:**
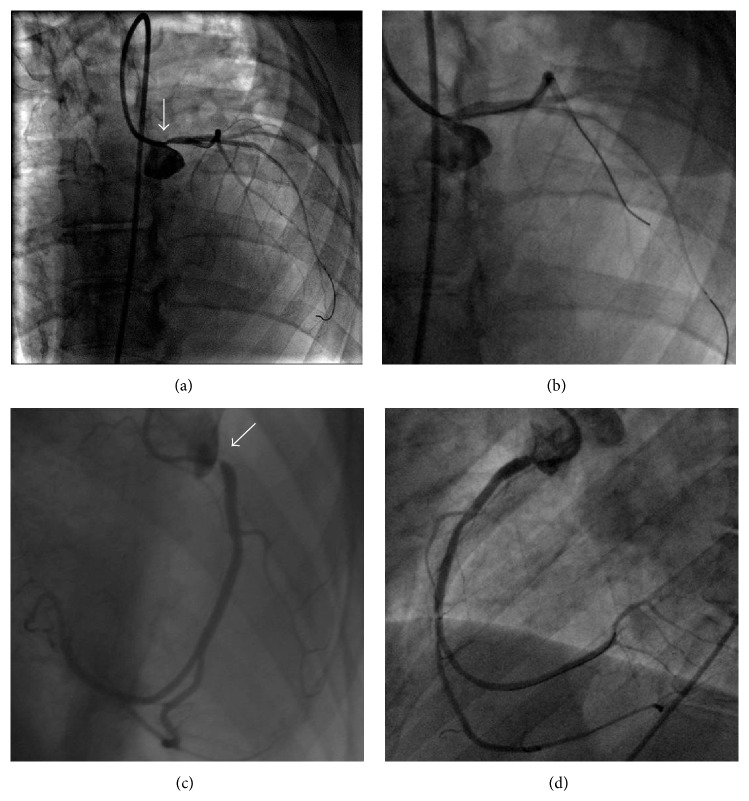
(a) Left coronary artery showing critical ostial left main stenosis (arrow). (b) Final result of the left main direct stenting with everolimus eluting stent. (c) Tight ostial lesion of the right coronary artery (RCA; arrow). (d) Final result after direct stenting of ostial RCA with sirolimus eluting stent.

Afterwards, she was admitted to our cardiology department for an invasive evaluation. Coronary angiography showed focal critical ostial left main (LM) stenosis (with damping pressure) and critical ostial right coronary artery (RCA) disease (Figure 2). Severe, diffuse calcification of the ascending aorta was also present. The cardiac catheterization showed severe aortic valve stenosis (peak-to-peak gradient 70 mmHg) and pulmonary artery hypertension (mean pulmonary artery pressure 35 mmHg, capillary wedge pressure 20 mmHg).

**Figure 3:**
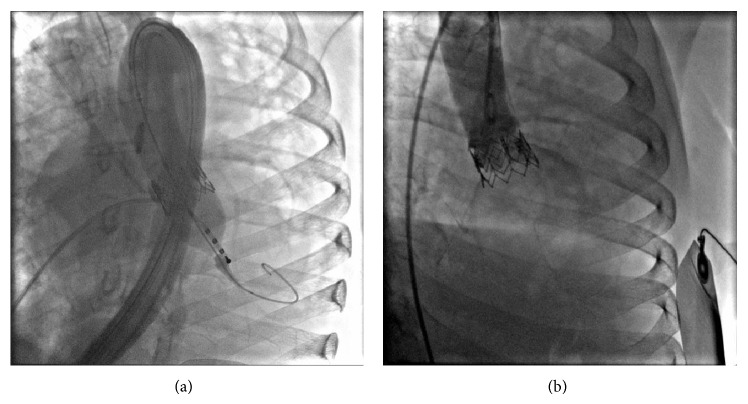
(a) Transcatheter aortic valve implantation (Edwards SAPIEN XT 23 mm). (b) Aortography after TAVI procedure.

Considering the high surgical risk due to severe restrictive lung disease, severe calcification of the ascending aorta, and postradiation thoracic adherence, the heart team planned a staged percutaneous approach. The patient underwent a successful coronary angioplasty with drug eluting stent implantation on ostial RCA and LM (sirolimus eluting stent 3.0 × 13 mm on RCA, everolimus eluting stent 3,5 × 12 mm on LM, Figure 2). According to angio-CT sizing, a 23 mm Edwards SAPIEN XT valve (Edwards Lifesciences, Irvine, CA) was selected for the procedure. One month later, she underwent TAVI procedure, performed through the right femoral artery (Figure 3).

After the procedure, the echocardiogram showed a markedly decreased transaortic valvular gradient (mean gradient 10 mmHg) with a mild periprosthetic regurgitation, improved left ventricular systolic function, and mild mitral regurgitation. The patient was discharged on aspirin, clopidogrel up to twelve months, ivabradine, and furosemide.

At 24 months' follow-up, she is in NYHA II functional class, fully carrying out normal daily activities. Echocardiogram confirms the good performance of the prosthetic valve and normal left ventricular systolic function.

## 3. Discussion

This case report describes a fully percutaneous treatment of the cardiovascular consequences of thoracic exposure to radiation therapy. This modern approach offered a concrete chance to this young woman, who otherwise should have undergone a very high risk surgical intervention.

The risk of long-term radiotherapy damage depends on the radiation dose and the field of exposure [8]. The pathogenesis of radiation-induced coronary artery disease (CAD) is complex and not yet fully understood; radiation exposure may act as an independent risk factor of arteriosclerosis. Radiation-induced valvular disease is uncommon [8]; valve lesions are diagnosed on average 11.5 years after radiation exposure and symptoms occur at least 5 years later [9].

The pathophysiology of radiation-induced valvular disease is not completely clarified: cellular injury, combined with pressure-related trauma, may cause valvular fibrosis and calcification [10, 11]: irradiation seems to trigger a degenerative process that lasts for years.

Most radiation effects are dose related and it is probable that modern techniques with lower radiation exposure and smaller treatment volumes may reduce these risks.

TAVI is a highly effective procedure for selected patients who are at high surgical risk. The lack of long-term outcomes limits the use of TAVI to the elderly. In this case, the young age represented a concern, overweighted by the prohibitive surgical risk.

## References

[1] Orzan F., Brusca A., Conte M. R., Presbitero P., Figliomeni M. C. (1993). Severe coronary artery disease after radiation therapy of the chest and mediastinum: clinical presentation and treatment. *British Heart Journal*.

[2] Stewart F. A., Seemann I., Hoving S., Russell N. S. (2013). Understanding radiation-induced cardiovascular damage and strategies for intervention. *Clinical Oncology*.

[3] Galper S. L., Yu J. B., Mauch P. M. (2011). Clinically significant cardiac disease in patients with Hodgkin lymphoma treated with mediastinal irradiation. *Blood*.

[4] Aqel R. A., Zoghbi G. J. (2006). Radiation therapy-related cardiovascular disease. *Journal of Heart and Lung Transplantation*.

[5] Thomas M., Schymik G., Walther T. (2010). Thirty-day results of the SAPIEN aortic bioprosthesis European outcome (SOURCE) registry: a European registry of transcatheter aortic valve implantation using the edwards SAPIEN valve. *Circulation*.

[6] Makkar R. R., Fontana G. P., Jilaihawi H. (2012). Transcatheter aortic-valve replacement for inoperable severe aortic stenosis. *The New England Journal of Medicine*.

[7] Kapadia S. R., Tuzcu E. M., Makkar R. R. (2014). Long-term outcomes of inoperable patients with aortic stenosis randomly assigned to transcatheter aortic valve replacement or standard therapy. *Circulation*.

[8] Hull M. C., Morris C. G., Pepine C. J., Mendenhall N. P. (2003). Valvular dysfunction and carotid, subclavian, and coronary artery disease in survivors of Hodgkin lymphoma treated with radiation therapy. *Journal of the American Medical Association*.

[9] Carlson R. G., Mayfield W. R., Normann S., Alexander J. A. (1991). Radiation-associated valvular disease. *Chest*.

[10] Brand M. D., Abadi C. A., Aurigemma G. P., Dauerman H. L., Meyer T. E. (2001). Radiation-associated valvular heart disease in Hodgkin's disease is associated with characteristic thickening and fibrosis of the aortic-mitral curtain. *Journal of Heart Valve Disease*.

[11] Katz N. M., Hall A. W., Cerqueira M. D. (2001). Radiation induced valvulitis with late leaflet rupture. *Heart*.

